# International Conference Addresses Preparedness for Emerging Strains of Pandemic Influenza

**DOI:** 10.3201/eid0201.960116

**Published:** 1996

**Authors:** Dominick A. Iacuzio

**Affiliations:** National Institutes of Health, Bethesda, Maryland, USA


For further information on WHO's rapid-response unit, contact

**Philippe Stroot**
Health Communications and Public Relations
World Health Organization
Geneva, Switzerland
Phone: 41-22-791-2535
Fax: 41-22-791-4858

### Rotavirus Vaccine Workshop Held

More than 125 participants from at least 15 countries attended the Fifth Rotavirus Vaccine Workshop at the Centers for Disease Control and Prevention (CDC) in Atlanta, Georgia, October 16-17, 1995.

Rotavirus has emerged as the most important cause of severe diarrhea in children worldwide. It is a problem not only in developing countries, where it kills an estimated 870,000 children each year, but also in the United States, where it remains the most important single cause of hospitalization or clinic visits for childhood diarrhea.

Moreover, although studies from many countries indicate that only four serotypes are predominant worldwide, some strains at every site studied cannot be serotyped. In some countries such as India, the diversity of strains is extensive. Further studies are needed to define the extent of cross-protection against these strains that is induced by the vaccine to determine whether additional antigens need to be included in vaccines for such areas.

This workshop included sessions on epidemiology, virology, pathogenesis and immunity, and vaccines currently being tested. Each session had numerous presentations by leaders in the field of rotavirus research. Researchers reported that several live oral rotavirus vaccines, based on animal strains of rotavirus combined with reassortant strains, have been tested in field trials in children. These appear to protect American children against rotavirus and are more efficacious against severe disease. These vaccines like natural protection, are not 100% protective so many investigators are exploring alternative approaches to vaccines such as the use of virus-like particles, native DNA, and microencapsulation of antigens.

No published volume of proceedings from the workshop is planned, but a supplemental issue of the *Journal of Infectious Diseases* scheduled for early 1996 will contain papers from the meeting.

The workshop was held under the auspices of the National Institutes of Health, Emory University School of Medicine, and the World Health Organization.

For further information, contact

**Roger I. Glass**
Global Program for Vaccines and Immunizations
World Health Organization
Geneva, Switzerland
Phone: 41-22-791-2698/2681
Fax: 41-22-791-4860

### International Conference Addresses Preparedness for Emerging Strains of Pandemic Influenza

An international meeting on pertinent issues related to recognizing, identifying, and controlling newly emerging strains of pandemic influenza was held in Bethesda, Maryland, December 11–13, 1995. The conference, "Pandemic Influenza: Confronting a Reemergent Threat," was sponsored by the National Institutes of Health, the University of Michigan, the Centers for Disease Control and Prevention, the Food and Drug Administration, the U.S.-Japan Cooperative Medical Science Program, and the World Health Organization.

Epidemic strains of influenza cause infections almost every year throughout the world because of continuous minor genetic changes in the virus. However, periodically a major change occurs, such as reassortment between mammalian and avian strains of the virus. These pandemic strains are novel to the human immune system and, therefore, can cause substantial disease worldwide. The conference concentrated on issues that would be crucial to controlling an influenza pandemic.

Plenary and workshop sessions examined the following topics: Can pandemics be predicted? What are the specific approaches for pandemic control? What are the advantages and limitations of vaccines and antiviral agents? The workshops also focused on factors contributing to the emergence of pandemic strains and various aspects of surveillance, such as the adequacy of current global surveillance structure for early identification of a pandemic strain, the use of virologic and epidemiologic surveillance once a strain is identified, and the rapid exchange of information globally. The following immunologic and molecular questions were addressed as well: What basic research advances would allow us to respond more rapidly after the next human pandemic strain is detected? Is the presence of novel influenza A virus in pigs a predictor of the next influenza pandemic? Is an H2 influenza virus the next human pandemic subtype or are H7 viruses equally possible? Also discussed were the practical issues of vaccine needs, production, and distribution.

Conference participants then reviewed international pandemic plans and the U.S. pandemic plan being prepared by the Federal Interagency Group on Pandemic Preparedness.

**Dominick A. Iacuzio**
Division of Microbiology and Infectious Diseases
National Institutes of Health
Bethesda, Maryland, USA

### Course Offered on Clinical and Pathologic Features of Emerging Infections

The Armed Forces Institute of Pathology (AFIP), Emory University, and the Centers for Disease Control and Prevention (CDC) are cosponsoring a course on emerging and reemerging pathogens. The course will be taught in Atlanta, Georgia from April 27 to May 1 and will discuss the epidemiology, clinical features, pathology, and pathogenesis of such diseases as plague, Lyme disease, Kaposi sarcoma, microsporidiosis, Buruli ulcer, ehrlichiosis, hantavirus pulmonary syndrome, and Ebola virus infection. Emerging drug resistance in pneumococci and other streptococcal infections will also be discussed.

The course is designed for pathologists, epidemiologists, infectious disease physicians, veterinarians, microbiologists, parasitologists, and others interested in the pathology as well as the emergence of infectious diseases. The course, to be held at the Emory Conference Center Hotel, will provide 38 hours of Category I CME credit and will consist of 32 hours of lectures with open discussion periods, 6 hours of glass and color slide review, and a visit to CDC laboratories. For more information, contact the course director, Center for Advanced Medical Education, AFIP, Washington, D.C. 20306-6000 (phone: 800-577-3749 or 301-295-7921; fax: 301-427-5001).

**C. Robert Horsburgh, Jr.**
Emory School of Medicine
Atlanta, Georgia, USA

### NASA Sponsors Symposium on Remote Sensing and Control of Insect-Transmitted Diseases

Health officials and disease control experts met November 28-30 in Baltimore, Maryland, for a symposium on the use of satellites to monitor and control insect-transmitted diseases.

Sponsored by the National Aeronautics and Space Administration (NASA) and the Third World Foundation of North America, the symposium was held to inform government officials from various countries of NASA's scientific and technologic capabilities for detecting, monitoring, and improving the control of diseases. Health ministers and medical directors from more than 20 countries, including Bangladesh, Belize, China, Ghana, Indonesia, Kenya, Malaysia, Nigeria, Peru, and Rwanda, attended.

The symposium featured discussions on the economics of disease surveillance, deforestation, and urbanization. The keynote address, "The resurgence of vector-borne infectious diseases as major public health problems in the 1990s," was given by Duane Gubler, director, Division of Vector-Borne Infectious Diseases, National Center for Infectious Diseases, Centers for Disease Control and Prevention (CDC). Participants also discussed possible joint activities between NASA and interested countries. Further information can be obtained from NASA's Office of Life and Microgravity Sciences, Washington, D.C., which manages the agency's global monitoring and human health research program in conjunction with the National Institute of Allergy and Infectious Diseases, National Institutes of Health, and CDC.

**Michael Braukus**
National Aeronautics and Space Administration
Washington, D.C., USA

**Raj Khanna**
Third World Foundation of North America
College Park, Maryland, USA

An international meeting on pertinent issues related to recognizing, identifying, and controlling newly emerging strains of pandemic influenza was held in Bethesda, Maryland, December 11–13, 1995. The conference, "Pandemic Influenza: Confronting a Reemergent Threat," was sponsored by the National Institutes of Health, the University of Michigan, the Centers for Disease Control and Prevention, the Food and Drug Administration, the U.S.-Japan Cooperative Medical Science Program, and the World Health Organization.

Epidemic strains of influenza cause infections almost every year throughout the world because of continuous minor genetic changes in the virus. However, periodically a major change occurs, such as reassortment between mammalian and avian strains of the virus. These pandemic strains are novel to the human immune system and, therefore, can cause substantial disease worldwide. The conference concentrated on issues that would be crucial to controlling an influenza pandemic.

Plenary and workshop sessions examined the following topics: Can pandemics be predicted? What are the specific approaches for pandemic control? What are the advantages and limitations of vaccines and antiviral agents? The workshops also focused on factors contributing to the emergence of pandemic strains and various aspects of surveillance, such as the adequacy of current global surveillance structure for early identification of a pandemic strain, the use of virologic and epidemiologic surveillance once a strain is identified, and the rapid exchange of information globally. The following immunologic and molecular questions were addressed as well: What basic research advances would allow us to respond more rapidly after the next human pandemic strain is detected? Is the presence of novel influenza A virus in pigs a predictor of the next influenza pandemic? Is an H2 influenza virus the next human pandemic subtype or are H7 viruses equally possible? Also discussed were the practical issues of vaccine needs, production, and distribution.

Conference participants then reviewed international pandemic plans and the U.S. pandemic plan being prepared by the Federal Interagency Group on Pandemic Preparedness.

